# Which methods are the most effective in enabling novice users to participate in ontology creation? A usability study

**DOI:** 10.1093/database/baab035

**Published:** 2021-06-22

**Authors:** Limin Zhang, Xingyi Yang, Zuleima Cota, Hong Cui, Bruce Ford, Hsin-liang Chen, James A Macklin, Anton Reznicek, Julian Starr

**Affiliations:** School of Information, University of Arizona, 1103 E. Second Street, Tucson, AZ 85705, USA; School of Information, University of Arizona, 1103 E. Second Street, Tucson, AZ 85705, USA; School of Information, University of Arizona, 1103 E. Second Street, Tucson, AZ 85705, USA; School of Information, University of Arizona, 1103 E. Second Street, Tucson, AZ 85705, USA; Department of Biological Sciences, University of Manitoba, 50 Sifton Road, Winnipeg, MB R3T 2N2, Canada; Curtis Laws Wilson Library, Missouri University of Science and Technology, 400 W. 14th Street, Rolla, MO 65409, USA; Information protected by Canada government, Agriculture and Agri-Food Canada, Ottawa, ON K1A 0C6, Canada; SLA Herbarium, University of Michigan, 3600 Varsity Drive, Ann Arbor, MI 48019, USA; Department of Biology, University of Ottawa, 30 Marie Curie Road, Ottawa, ON K1N 6N5, Canada

## Abstract

Producing findable, accessible, interoperable and reusable (FAIR) data cannot be accomplished solely by data curators in all disciplines. In biology, we have shown that phenotypic data curation is not only costly, but it is burdened with inter-curator variation. We intend to propose a software platform that would enable all data producers, including authors of scientific publications, to produce ontologized data at the time of publication. Working toward this goal, we need to identify ontology construction methods that are preferred by end users. Here, we employ two usability studies to evaluate effectiveness, efficiency and user satisfaction with a set of four methods that allow an end user to add terms and their relations to an ontology. Thirty-three participants took part in a controlled experiment where they evaluated the four methods (Quick Form, Wizard, WebProtégé and Wikidata) after watching demonstration videos and completing a hands-on task. Another think-aloud study was conducted with three professional botanists. The efficiency effectiveness and user confidence in the methods are clearly revealed through statistical and content analyses of participants’ comments. Quick Form, Wizard and WebProtégé offer distinct strengths that would benefit our author-driven FAIR data generation system. Features preferred by the participants will guide the design of future iterations.

## Introduction

Phenotypes are ‘the set of observable characteristics of an individual resulting from the interaction of its genotype with the environment’ (Oxford Dictionary), such as leaf length or eye color. Phenotypic characters are paramount for describing species, studying function and understanding organismal evolution, but only a very small amount of historical and newly published data are represented with clear semantics via ontological annotations. The lack of such computable data is due to the high cost of manual annotation, incomplete phenotype ontologies and high inter-curator variations (1, 2).

An alternative approach to produce findable, accessible, interoperable and reusable (FAIR) data (3) is to directly enable authors to write their scientific findings in a semantically explicit language by employing ontologies in their writing workflow. We presented this idea, author-driven computable data and ontology development, to the US National Science Foundation and were awarded funding to investigate effective tools and incentives for the authors to adopt this new workflow (4). Currently, for many biological ontologies developed under the OBO (Open Biological and Biomedical Ontology) foundry framework (5), new term proposals have to go through a lengthy vetting process by ontology engineers before they are approved or rejected. This significantly hinders the construction and use of the ontologies. We investigated a different approach, where authors can add terms to the ontology as needed and where the life span of the term in the ontology is determined by other competing terms and their respective frequencies of usage by the community.

This approach has the following advantages: (i) authors interact with the ontology frequently and this increases their familiarity with it; (ii) authors contribute terms to the ontology, making them more likely to think of it as ‘their’ ontology, increasing authors’ buy-in; (iii) with increased familiarity, stronger buy-in and frequent usage of the ontology, authors can quickly spot issues in the ontology and (iv) with most of the knowledge acquisition work now completed by authors, ontology engineers can be left to focus on solving issues and designing useful patterns that in turn would make authors’ contributions more effective and efficient. We believe this approach will address the well-known issues of current ontology construction and use, namely, knowledge acquisition bottleneck (6), incomplete coverage of ontologies (1, 2) and misuse of terms in an ontology due to term label and definition issues (1). We believe that an author’s direct contribution to phenotype ontologies is needed, because phenotypic characters are highly complicated and their interpretations are often subtle and require substantial knowledge on the specific taxon in question. Upper-level ontologies for phenotypic ontologies are largely in place. For example, Basic Formal Ontology and a fair number of anatomy ontologies for different taxonomic groups like plants (Plant Ontology, 35) and hymenopterans (HAO Ontology, A Gross Anatomy Ontology for Hymenoptera; 40) are now available. These existing ontologies can be leveraged in developing ontologies needed to support authors.

**Figure 1.**
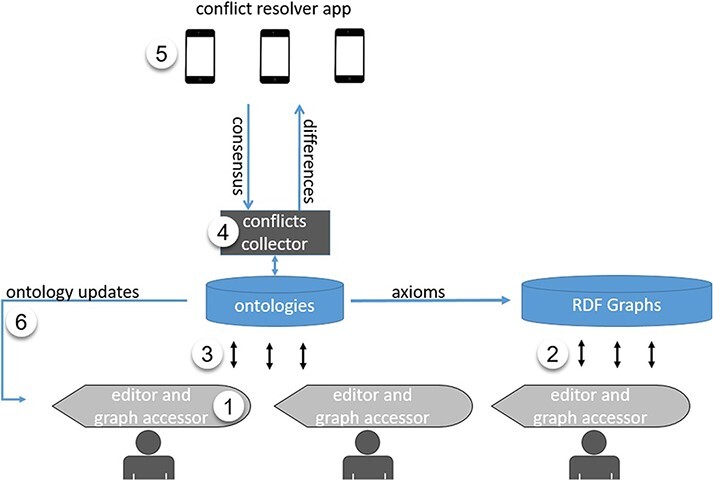
System design for the author-driven computable data and ontology development platform.

The complete prototype system (Figure 1) of author-driven computable data and ontology development consists of (i) an editor software application for authors to document their phenotypic characters by adding and using terms in ontologies; (ii) a semantic module that formats phenotypic characters as Resource Description Framework (RDF) named graphs and aggregates the graphs daily from different authors and for different taxa; (iii) an ontology Application Programming Interface (API) that updates the ontologies with authors’ input while working in the editor; (iv) an ontology scanner that scans and collects ‘conflicts’ in current ontologies; (v) a mobile application that presents conflicts for domain experts to vote on and resolve and (vi) term usage and provenance tracking modules that track and document all term usages and changes made to the ontology.

One key component of the system presented in Figure 1 is a user interface that allows an author to add terms and relations to a phenotype ontology. Because we hope to attract a broad group of biology authors to use this new ontology-based authoring environment, we need to find user interfaces that are preferred and easily accepted by them.

Usability as the core of product design has been broadly recognized in computer engineering and HCI fields (7, 8, 9, 10). Usability refers to ‘the question of how well users use that functionality’ and ‘how well’ can be systematically approached, improved and evaluated (possibly measured) through five attributes: learnability, efficiency, memorability, errors and satisfaction (11). According to the latest International Organization for Standardization (ISO) 9241-11(Technical Committee ISO/TC 159 Ergonomics 2018), usability is ‘the extent to which a system, product, service can be used by specified users to achieve specific goals with effectiveness, efficiency, and satisfaction in a specified context of use’. This definition takes into account the effectiveness, efficiency (including learnability and memorability in Nielsen’s term) and user satisfaction offered by the interaction between users and the product. In this paper, we report a usability study on four different user interfaces that can be employed by end users (as opposed to ontology engineers) to add terms and relations to an ontology in order to distill desirable features authors would use in the new authoring workflow.

Evaluation of ontology editors has been approached with different methods and criteria, but few have evaluated an editor’s usability as defined above while using a reasonably large number of users and real ontological tasks. The few studies involving expert users and novice users usually had a small number of participants. In searching for insights to design intuitive user interfaces for common biology authors to participate in phenotype ontology construction, we conducted two usability experiments to compare four web-based ways of adding terms to an ontology, two of which were developed by the authors, while the other two are well-known tools already in wide use by ontology engineers and/or curators. The four interfaces were as follows: (i) Quick Form, a simple web form that is connected to the ontology; (ii) Wizard, a list of questions that guide the user through a process of adding terms, synonyms, part-of and has-part relations to the ontology; (iii) Wikidata, an open knowledge-based used by some biological ontologies based on the familiar Wiki platform (12), and (iv) WebProtégé, the web version of the well-known Protégé Ontology Editor (13). These four methods were selected or developed to cover the spectrum of the ways a user could add a term to an ontology via a text-based interface. Innovative graph-based ontology or knowledge map construction methods were excluded in this study.

Our goal was to understand users’ preferences toward different interfaces and different features of these interfaces by answering four research questions: (i) what is the relative effectiveness of the four methods? (ii) what is the relative efficiency of the four methods? (iii) what is the user satisfaction with each of the four methods? and (iv) what features contributed to the above results?

## Methods and data

Two experiments were conducted. One was a controlled experiment with 36 student participants recruited from the School of Information, University of Arizona, during the spring semester of 2019. The other was a usability evaluation session using a modified think-aloud protocol with three botanists in May 2019. Participants used four different methods to add terms to ontologies and commented on their experiences and the interfaces.

We elaborate experiment designs, followed by evaluation metrics, and data analysis methods.

### Four different methods

All. The four interfaces cover major text-based user interfaces through which a term could be added to an ontology and they make different assumptions about the user.

#### Quick Form

**Figure 2.**
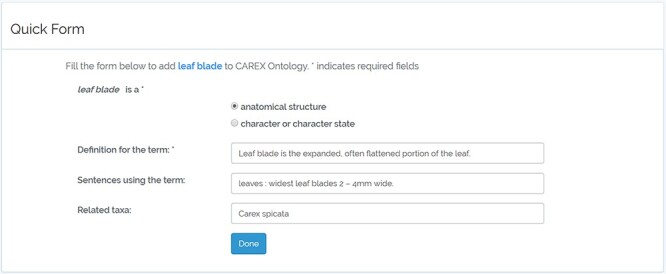
A screenshot of Quick Form.

Quick Form is a simple HTML form that collects basic information about the term to be added to the ontology. This interface assumes no ontology-related knowledge in its users. Anyone with basic web use experience can use it. The interface provides Javascript and server-side form validation and is potentially customizable and extensible in many ways. A screenshot of Quick Form is shown in Figure 2, and a YouTube video of how to use Quick Form with ‘leaf sheath’ as an exemplar can be found at the following website: https://www.youtube.com/watch?v=jNyglaeDz1E&t=8s.

#### Wizard

**Figure 3.**
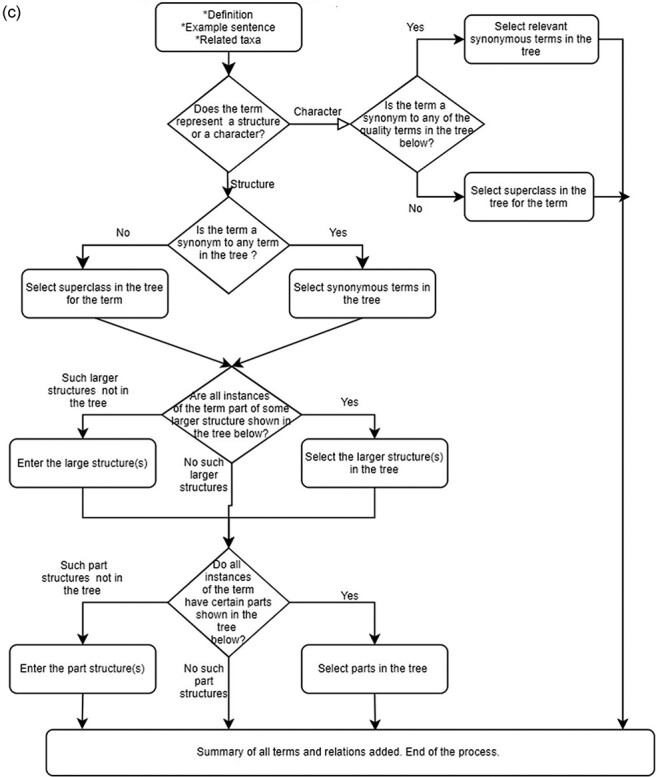
(continued)

‘A wizard presents a series of steps or conditions that the user needs to complete in order to accomplish a goal’ (Babich, 2017). Wizards, as applied in software, simplify the task and reduce the burden of decision-making for the user. Our Wizard organizes decisions the user needs to make into a decision tree to add a term and its synonym, meronyms and holonomies to the ontology. Based on the answer, it directs the user to the appropriate next step in the process. This interface assumes the user has rich experience with web interface gadgets and basic knowledge of hierarchical structure in a taxonomy, in addition to synonyms and parts of relations. If the wizard approach proves useful, the Wizard interface can be extended to accommodate other ontological constructs and design patterns beyond the part of relations. Figure 3a and b shows a few screen captures of Wizard, and the following YouTube video demonstrates how to use Wizard with ‘leaf sheath’ as an exemplar: https://www.youtube.com/watch?v=6oMmKp4G1Js.

#### Wikidata

Wikidata is ‘a free and open knowledge base that can be read and edited by both humans and machines’ (Wikidata, 2021) (15). It ‘acts as central storage for the structured data of its Wikimedia sister projects, including Wikipedia’ (Wikidata main page) and many other projects not associated with the Wikimedia Foundation (Wikidata, 2021) (15). Because the underlying data model of Wikidata is RDF recommended by the W3C for the Semantic Web, many ontologies are also hosted in Wikidata as structured data. Because Wiki-based platforms have also been recognized as a useful platform for building community ontologies (16, 17, 18), we included Wikidata in this study.

**Figure 4.**
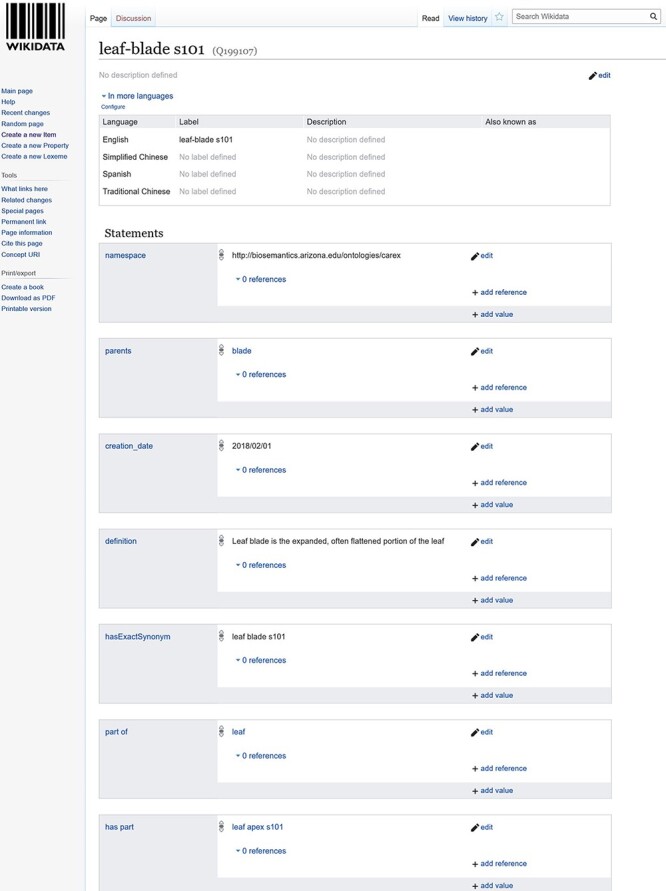
A screenshot of Wikidata IDs of properties interface.

Ontologies on Wikidata are presented as pages. Pages are all parallel to each other. This means, even though there may be pages of ontology A vs. pages of ontology B at the conceptual level, there are no structural or visual constructs on Wikidata that make such distinctions visible to a user. A page holds information for one term, which includes term ID, labels of the term in different languages and cross-references to other Wikimedia resources. Users can add an unlimited number of statements related to the term on a page (Figure 4). A statement consists of the term, a property and a value to the property, and it is a way to add RDF properties to a term. Wikidata differentiates entities (i.e. classes) and properties by giving them different initial letters in their IDs: IDs of entities start with Q, while IDs of properties start with P. For example, the property ‘part of’ has ‘P791’ as its property ID, and the term leaf-blade has an ID Q91238383 (Figure 4). Entities and properties are all defined by users and approved by the Wikidata editorial team. In this experiment, we used the sandbox version of Wikidata at https://test.wikidata.org/, which provides exactly the same functionality as the production site but without editorial control. Figure 4 is a screen capture of a term page in Wikidata. The demo used in the experiment that shows how to use Wikidata is found at https://youtu.be/5tGV7VzABv8.

#### WebProtégé

**Figure 5.**
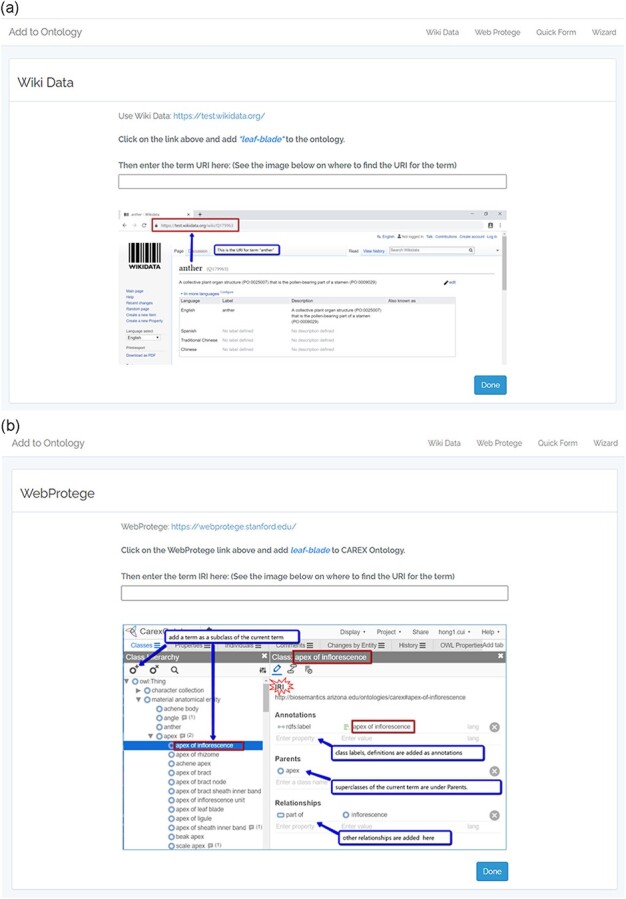
A screenshot of the WebProtégé classes interface.

WebProtégé is the web version of the well-known Protégé ontology editor. Compared to the desktop Protégé, it provides a highly customizable editing interface and a variety of productivity-enhancement features. It offers a simplified user interface by moving advanced semantic axiom editing out of the way of novice users. WebProtégé includes a set of predefined tabs and forms that contain the most commonly used functions For example, the predefined Classes Tab enables users to browse and edit the class hierarchy and the properties of classes; the Properties Tab provides access to the details of the properties in the ontology and allows the user to add any relationship properties and associated values (19, 20). A screen capture of the class user interface is shown in Figure 5. WebProtégé 4.0.0-beta-1 was used in the experiment. The demo used in the experiment that shows how to use WebProtégé is available at https://www.youtube.com/watch?v=SxGg_b8FRvM.

Quick Form and Wizard can be seen as user interfaces that mediate other applications to their backend ontologies. They can be embedded in these applications directly. WebProtégé and Wikidata, on the other hand, expose one or more ontologies in their entirety to the user. To construct the new workflow we envisioned, both could have a role in involving community participants in the FAIR data generation and ontology backbone construction. For example, authors may find Quick Form more likable as they hide the complexity of a full ontology, while members who have a strong interest in building the information infrastructure for the community may see Wizard as a more useful tool.

Other useful tools were reviewed by the project team but not selected for experiments due to time constraints. These tools are described in the ‘Related work’ section.

### Experimental design

#### Controlled experiment with graduate student participants

**Figure 6.**
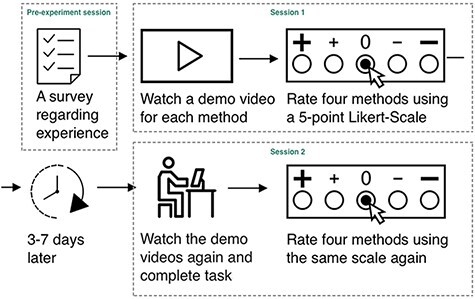
(a) The experiment website entry page for Wikidata. (b) The experiment website entry page for WebProtégé.

The controlled experiment was conducted remotely on Zoom, with the recording of participants’ on-screen activities. Additional user activities (e.g. click a button) were recorded by the built-in logging modules of Quick Form and Wizard, both of which were developed by the authors. To record the time spent by users on the external tools Wikidata and WebProtégé, we created an entry page for either tool on our experimental website. The entry page allowed us to record the time the user began a task by clicking on the link to Wikidata or WebProtégé and the time when the user completed the task by clicking on a ‘Done’ button. The difference between the two timestamps was the time the user spent on the respective tool. Figure 6 shows the entry page for Wiki Data (Figure 6a) and WebProtégé (Figure 6b).

#### Participants

Forty participants in a required graduate-level course in the School of Information at the University of Arizona were invited to take part in the experiment. Thirty-six accepted the invitation, but only thirty-three correctly followed instructions and completed the experimental sessions. Participants were assigned arbitrarily into one of the four groups following a Latin Square design, where each group would use the four methods in a different order as listed below

Group 1 (nine participants): Method order of use: A, B, C, D

Group 2 (eight participants): Method order of use: B, C, D, A

Group 3 (nine participants): Method order of use: C, D, A, B

Group 4 (seven participants): Method order of use: D, A, B, C

Here A stands for Quick Form; B, Wikidata; C, WebProtégé and D, Wizard.

Gender and age information were not collected in the experiment because (a) there is no evidence that within graduate students, gender or age affects an individual’s preference over controlled vocabulary building tools, and (b) these are identifiable information. However, we estimated that participants’ gender distribution was roughly 24 females and 9 males, and the age range was 25–45 years.

#### Experimental procedure

**Figure 7.**
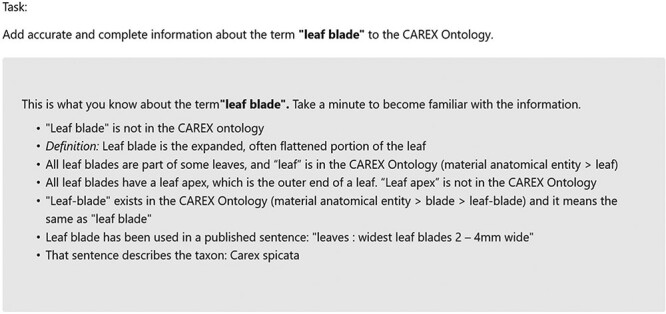
Experimental procedure.

The experiment consisted of a pre-experiment session and two activity sessions. A schematic representation of the experimental procedure is shown in Figure 7.

In the pre-experiment session, participants remotely filled out a survey consisting of four questions regarding their experience with controlled vocabulary editors and wikis. These questions are listed in Table 1.

**Table 1.** Pre-experiment survey questions

Questions	Answers				
1. How familiar are you with controlled vocabulary?	Extremely familiar	Very familiar	Moderately familiar	Slightly familiar	Not familiar
2. What is your experience with controlled vocabulary editors?	Frequent user	Used at least once	Heard of them but never used	Never heard of them	
3. How confident do you feel using controlled vocabulary editors?	Very confident	Moderately confident	Somewhat confident	Slightly confident	Not at all confident
4. How familiar are you with each of the following platforms? Wiki Wikidata Protege Ontology Editor	I am an expert editor on the platform	Can edit anything on the platform with little help	Edited at least one thing on the platform	Heard of it but never edit anything on it	Never heard of it

After completing the pre-experiment questionnaire, participants were scheduled to take part in the first activity session. Here, participants watched a 3–6-minute video tutorial for each method in the order defined for their group. Each video explains how to use a given method to add terms, synonyms or part of/has part relations, when relevant to the ontology. After watching each video, participants completed a web-based questionnaire that ranks each method in terms of its usability, its support for recording the full semantics of a term and the user’s confidence level if asked to use the method to complete a task (Table 2). All videos were available to the participants while they completed the questionnaire.

**Table 2.** User preference survey questions (for both activity sessions)

Questions
1. Please rank each editor based on their level of difficulty (1 = easiest to use, 4 = hardest to use).
2. It was easy for me to rank the editors based on the perceived difficulty level (1 = strongly agree, 2 = somewhat agree, 3 = neither agree nor disagree, 4 = somewhat disagree, 5 = strongly disagree).
3. (If selected 4 or 5 for question 2) Explain your difficulty in ranking Wizard, Quick Form, WebProtégé, Wiki Data on their ease of use.
4. Please rank each editor based on their helpfulness for fully documenting terms and relationships (1 = least helpful, 4 = most helpful).
5. It was easy for me to rank the methods based on their helpfulness for fully documenting terms and relationships (1 = strongly agree, 2 = somewhat agree, 3 = neither agree nor disagree, 4 = somewhat disagree, 5 = strongly disagree).
6. (If selected 4 or 5 for question 5) Explain your difficulty in ranking Wizard, Quick Form, WebProtégé, Wiki Data on their support for fully documenting terms and relationships.
7. How confident do you feel now using each of the four methods to add new terms and relationships? (1 = not at all confident, 4 = completely confident)

Participants were scheduled to take part in the second activity session 3–7 days later. In this session, participants watched the videos again and completed a hands-on task using each of the four methods to add new terms, synonyms and/or properties (e.g. part of/has part) related to ‘leaf blade’ to the CAREX Ontology, developed in consultation with the Plant Ontology Consortium (http://planteome.org/). After finishing the task, participants responded to the same questionnaire (Table 2) as in the first activity session. The CAREX Ontology contained over 2000 phenotype terms and some properties describing the plant genus *Carex* L., also known by the common name of ‘sedges’. This was the ontology created for the other project and the development of Measurement Recorder, a software tool for species descriptions that produce computable phenotypes (Cui *et al.* 2020).

**Figure 8.**
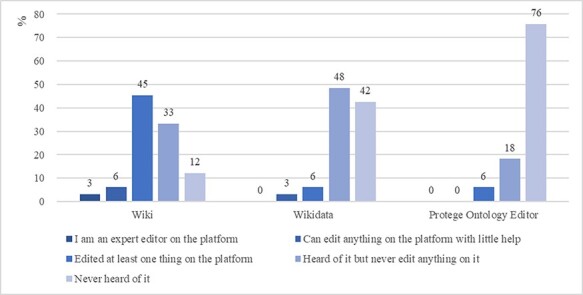
A part of the experiment instructions showing the information about ‘leaf blade’. The participants were asked to ‘Add accurate and complete information about the term’ to an ontology.

In both activity sessions, participants followed instructions laid out in a HTML page relevant to their session and group. For example, the instruction for the second activity session of Group 1 can be found at http://shark.sbs.arizona.edu/experiments/Session2Group1.html. The only difference in the instructions for different groups was the order in which the methods should be used. The instruction for the second activity session included the information about ‘leaf blade’, as shown in Figure 8. The participants were asked to ‘add accurate and complete information about the term’ to a phenotype ontology. This task involves adding typical phenotypic information such as new terms, new synonyms, part-of and has-part relationships to the ontology.

#### Think-aloud session

Three botanists at different career stages who are working on the our project participated in a group think-aloud session and added the term ‘leaf blade’ to the ontology using the four methods. One botanist worked directly on the user interfaces, while projecting his screen to a large white screen for the other two botanists to see. All three talked about what they thought of each of the screens that were displayed during the process.

### Measurements

Effectiveness refers to ‘the accuracy and completeness with which users achieve specified goals’ (22), and it is measured through the correctness and completeness of the tasks performed by the participants under different conditions. Efficiency is ‘the resources (time, human effort, money, and materials) used in relation to the results achieved’ (22). In this study, it was measured through task completion times under different conditions. Satisfaction concerns ‘the extent to which the user’s physical, cognitive, and emotional responses that result from the use of a system, product or service meet the user’s needs and expectations’ (22) was evaluated by analyzing participant responses to the questionnaire and examining the video recordings of their screen activities.

### Data analysis methods

Owing to relatively small sample sizes and the use of ordinal and categorical data, only nonparametric statistical tests were applied in data analyses.

The Friedman rank sum test is designed to detect differences in *N* treatments across multiple repeated tests, where *N* > 2. We used this test to detect differences in individual subtask completion rates, participant perceived difficulty, helpfulness and confidence ranks, across all four methods. We then used a Wilcoxon signed-rank test as a follow-up to detect the difference between any two methods. When examining task completion time difference, only Wilcoxon signed-rank tests were used for pairwise comparisons because only nine participants completed all tasks, a sample size too small for a Friedman rank sum test.

Cochran’s Q test is similar to a Friedman rank sum test, but it is designed for binary observations. Consequently, we used Cochran’s Q test to compare the differences in success (1) and failure (0) of participants in completing each subtask (defined in the ‘Effectiveness of the four methods’ section).

Fisher’s exact test is an alternative to a Chi-square test for categorical data, and it should be used when expected values in any of the cells of a contingency table are below 5. We used Fisher’s exact test to any correlation between post-task confidence level in using Wikidata and past experience with Wiki.

The Kruskal–Wallis test is designed to determine whether samples originate from the same distribution, and it can be used to compare two or more independent samples of equal or different sample sizes. This test was appropriate for detecting differences in task completion times among the four different orders in which each method was followed by participants during testing, given small sample sizes and the unequal task completion by participants using a tool sequence. The samples were independent because one method was used as the first, second, third and fourth tool by different and independent participants.

## Results and analyses

### Results from the controlled experiment

#### Pre-experiment survey results

Through the pre-experiment survey, we found that most of the participants were not new to the concept of controlled vocabulary. When it comes to controlled vocabulary editors, 6% of the participants reported that they had never heard of such editors, and 94% of the participants had either heard of or had used an editor before. But none of the participants were very confident in their ability to use an editor: a vast majority of the participants were either slightly confident or not confident at all (45% and 36%, respectively), with 6% reporting they were moderately confident (Table 3). These responses roughly correspond to the profile of biologists who took the ‘Biologists Attitude Towards Controlled Vocabularies’ survey recently conducted as part of our Other project (manuscript under preparation): among the 91 survey respondents, 13% never heard of ‘controlled vocabularies’, 47% knew the concept of controlled vocabulary and 13% created a controlled vocabulary.

**Table 3.** Pre-experiment survey results

Questions	Response (%)				
1. How familiar are you with controlled vocabulary?	Extremely familiar 3	Very familiar 6	Moderately familiar 52	Slightly familiar 39	Not familiar 0
2. What is your experience with controlled vocabulary editors?	Frequent user 3	Used at least once 33	Heard of them but never used 58	Never heard of them 6	
3. How confident do you feel using controlled vocabulary editors?	Very confident 0	Moderately confident 6	Somewhat confident 12	Slightly confident 45	Not at all confident 36

**Figure 9.**
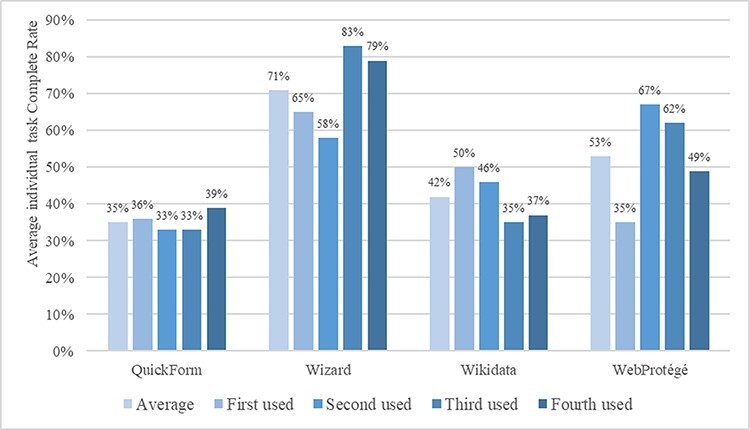
Participants familiarity with the Wiki, Wikidata and Protégé platforms (%).

In terms of Wiki, Wikidata and Protégé experiences, 45% of the participants had edited at least one thing on a Wiki, while 90% or 94% of the participants had never edited anything on Wikidata or Protégé, respectively (Figure 9). This set of data shows that Wikidata and Protégé were new to most participants, although some had experience using other wiki platforms This profile of the tool usage experience roughly matches what we would expect of common biology authors.

#### Effectiveness of the four methods

To assess the effectiveness of each method, we compared the correctness and completeness of the information participants added to the ontology.

Based on the information provided in Figure 9, ‘leaf blade’ was a synonym of ‘leaf-blade’, and ‘leaf-blade’ was found in the ontology. This meant that all information given for ‘leaf blade’ should be applied to ‘leaf-blade’, and ‘leaf blade’ should be added as a synonym to ‘leaf-blade’. Therefore, the complete and correct way of completing the task is to complete all nine subtasks listed below

1. Classifying leaf blade as an anatomical structure
2. Adding a definition for leaf-blade
3. Adding a relationship ‘part of leaf’ for leaf-blade
4. Adding a relationship ‘has part leaf apex’ for leaf-blade
5. Adding a new term leaf apex
6. Adding a definition of leaf apex
7. Adding a synonym ‘leaf blade’ for leaf-blade
8. Adding a sentence for leaf-blade
9. Adding a taxon for the sentence

Table 4 shows the task breakdown and task completion rates of the participants using the four methods, regardless of the method order. We define

**Table 4.** Effectiveness comparison of the four methods using task completion rates

Task		Quick Form (*n* = 33)	Wizard (*n* = 33)	Wikidata (*n* = 33)	WebProtégé (*n* = 33)	*P*-value
Task completion rate		0	27.3	0	0	
Average individual subtask completion rates^a^		35.4	71.4	42.1	52.6	Friedman ≤0.001
Task breakdown^b^	1. Classifying ‘leaf blade’ as an anatomical structure	87.9	93.9	100.0	93.9	C’s Q 0.225
	2. Adding a definition for ‘leaf-blade’	90.9	97.0	69.7	100.0	C’s Q ≤0.001
	3. Adding a relationship ‘part of leaf’ for ‘leaf-blade’	0.0	69.7	60.6	72.7	C’s Q 0.339^c^
	4. Adding a relationship ‘has part leaf apex’ for ‘leaf-blade’	0.0	63.6	45.5	57.6	C’s Q 0.101^c^
	5. Adding a new term ‘leaf apex’	0.0	63.6	42.4	60.6	C’s Q 0.068^c^
	6. Adding a definition of leaf apex	0.0	63.6	12.1	0.0	C’s Q ≤0.001^c^
	7. Adding a synonym ‘leaf blade’ for ‘leaf-blade’	0.0	48.5	63.6	75.8	C’s Q 0.022^c^
	8. Adding the sentence for leaf-blade	78.8	84.8	0.0	66.7	C’s Q ≤0.001
	9. Adding a taxon for the sentence	57.6	60.6	3.0	0.0	C’s Q ≤0.001

a Friedman rank sum test was used to determine the difference in individual task completion rates among four methods, and pairwise comparisons of the methods were conducted as well.

b Cochran’s Q test was conducted to determine the difference in the completion rate of each subtask.

c Quick Form was excluded from the Cochran’s Q test.

Task completion rate = number of participants who completed the task/total number of participants.

Individual subtask completion rate = number of completed subtasks by a participant/total number of subtasks (= 9)

Table 4 shows that when using Wizard, 27.3% of the participants accurately completed all subtasks; in contrast, no participants completed all subtasks when using any other method. On subtasks, the average individual subtask completion rates were 35.4% (Quick Form), 42.1% (Wikidata), 52.6% (WebProtégé) and 71.4% (Wizard). The differences in individual subtask completion rates were statistically significant among the four methods (Friedman rank sum test, *P* < 0.001). The follow-up pairwise comparison between any two methods using the Wilcoxon signed-rank test showed that Wizard had significantly higher individual subtask completion rates than other methods (*P* < 0.016). Wikidata and WebProtégé had similar performance, while Quick Form’s completion rates were the lowest (*P* < 0.0014).

Table 4 shows the ratio of the participants who completed each of the subtasks in the ‘task breakdown’ section. To compare the differences in success and failure in completing each subtask, Cochran’s Q tests were conducted with *P*-values presented in Table 4. Note, for subtasks 3–7, Quick Form was excluded from the test because its interface did not include an input box for these subtasks. Looking at the set of subtasks, participants using Quick Form had a relatively high completion rate on subtasks that are visually presented in the form, including classifying leaf blade as an ‘anatomical structure’, and adding definition, sentence and related taxon, but they had zero completion rates on all other subtasks.

When using QuickForm, we had different expectations as to what the different groups of participants might enter in the ‘Definition for the term’ input box (Figure 2). Since Group 1 participants used QuickForm as their first tool, we only expected them to enter the definition for a term in the input box. However, for other groups, we expected that the request to ‘[a]dd accurate and complete information about the term’ would influence participants who had used other tools to include additional information such as ‘synonym relations’ and ‘part-of’ statements to the input box. No participant did so, suggesting that an effective QuickForm design should include multiple input boxes requesting specific information.

When using Wikidata, the participant’s average completion rate on each of the subtasks was lower than when using WebProtégé or Wizard. Notably, among the five subtasks with statistically significant performance differences (Table 4, in bold), WebProtégé earned the highest scores for two subtasks, while Wizard scored the highest on the other three. Further, Wizard was the only method for which all subtasks were correctly completed by some participants.

Overall, the results in Table 4 suggest that Wizard offers the greatest effectiveness on task completion, followed by WebProtégé, Wikidata and Quick Form.

Recall that participants were assigned to one of the four groups based on a Latin square design to account for ordering and learning effects. Participants used the four methods in different orders to complete the same task. We expect that later in the session, participants would become more familiar with the task information (leaf blade and related information). Taking the order of method usage information into consideration, Figure 9 shows the average individual task completion rates by method and by order of use.

**Figure 10.**
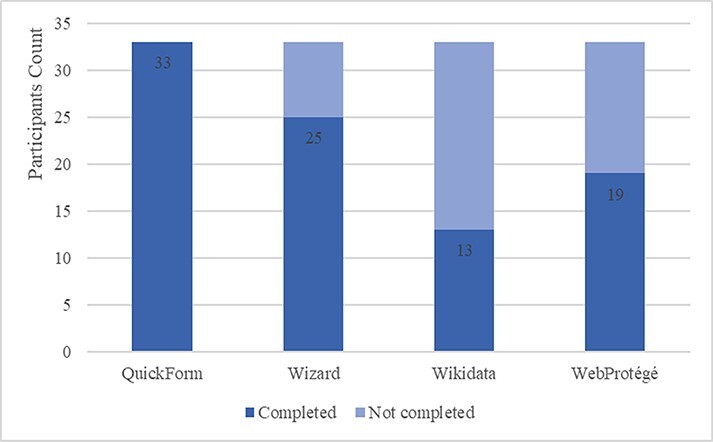
Average individual task completion rates (%) by method and by order of use.

The results in Figure 10 confirm the earlier conclusion on overall effectiveness of the four methods. They further reveal that when the participants became more familiar with the task information, the advantage of Wizard became clearer—the average individual task completion rates reached 80% and greater when Wizard was the third or fourth method used. In contrast, the effectiveness of WebProtégé decreased when used later in the experiment, despite a dramatic increase in effectiveness when used as the second method in experiments. This increase followed by a decline suggests a learning and fatigue effect were both in play. Even with the fatigue effect, the completion rate of using WebProtégé as the last method was higher than when used as the first method. The effectiveness of Wikidata seems to follow a downward trend, suggesting the fatigue effect overwhelmed the learning effect. Quick Form’s performance was relatively consistent throughout, regardless of the order of use. This can be explained by its simple interface such that neither the learning effect nor the fatigue effect was relevant.

#### Efficiency of the four methods

The efficiency of methods is measured by the time participants spent on the task using each method. Because the instructions for the experiment asked participants to stop when they had worked on the task for 5 minutes, this part of the analysis separates participants who reported that they completed the task within the time frame from those who reported that they did not complete the task.

**Figure 11.**
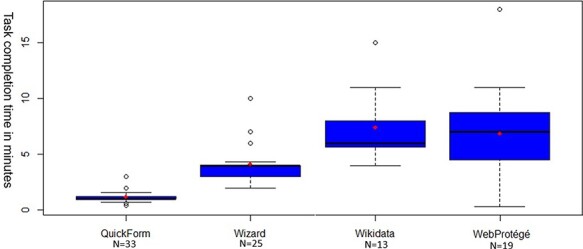
Self-reported task completion count of the four methods.

Figure 11 shows that all 33 participants completed the task using Quick Form, followed by Wizard (20), WebProtégé (23), and Wikidata (24). Once again, this confirmed the finding reported above on the effectiveness of each method.

**Figure 12.**
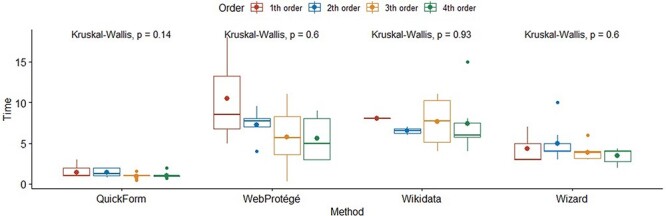
Boxplots of task completion times for participants who completed the experiment using a particular method. Boxes cover 50% of the data values ranging between the 25th and 75th percentiles, and lines show 90% of values within the 5th and 95th percentiles. Lines within boxes represent medians. Outlying values are indicated by the small ‘o’.

Figure 12 shows the boxplots of the task completion times of the participants who reportedly completed the task. Because only nine participants completed all tasks, a Friedman rank sum test was not used to compare the completion time, instead, Wilcoxon signed-rank tests were conducted among pairs of the four methods (Table 5). Results suggest that Quick Form was the most efficient, followed by Wizard, Wikidata and WebProtégé. Participants spent a significantly longer time on the task using Wikidata or WebProtégé than using the other two methods. After removing the outliers shown in Figure 12, the mean time spent using Wikidata (6.76 minutes) is more than five times the time spent using Quick Form (1.26 minutes), although the task completion rates for the two were comparable (42% vs. 35%, Figure 10). The mean time spent using WebProtégé (6.24 minutes) is about five times the time spent using Quick Form, while the task completion rate of the former is only about 50% more (53% vs. 35%, Figure 10). Wizard’s time cost (3.51 minutes) and task completion rate (71%) are both about twice those for Quick Form.

**Table 5.** Wilcoxon signed-rank tests on task completion time between pairs of methods

	Quick Form	Wizard	Wikidata
Wizard	*n* = 25 *P* < 0.001		
Wikidata	*n* = 13 *P* < 0.05	*n* = 11 *P* < 0.05	
WebProtégé	*n* = 19 *P* < 0.001	*n* = 17 *P* < 0.05	*n* = 10 *P* = 0.767

**Figure 13.**
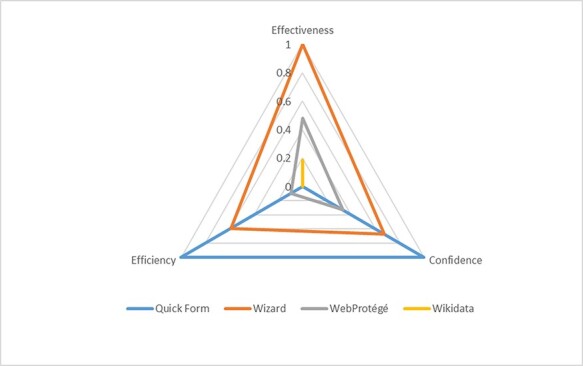
Boxplots of task completion times by method and by order of use.

Kruskal–Wallis tests for task completion time by method and order of use were not significant. Figure 13 summarizes this finding. This suggests that the four methods were so different that the experience of using one method does not help improve the efficiency of another. This confirms that our choice of methods to compare was good.

#### User satisfaction for the four methods

Results reported in this section are based on participants’ responses to the survey questions shown in Table 2. The four methods are ranked based on their difficulty level in usage, their helpfulness in recording term semantics, participants’ confidence in their ability of using the method and their responses to two open-ended questions ‘What specific features did you like about this method? Why?’ and ‘What did you not like about this method? Why?’.

##### Results based on response to the ranking questions

Table 6 presents the mean ranks of difficulty, helpfulness and confidence for the four methods as perceived by the participants after watching the demonstration videos in the first activity session and after completing the hands-on task after the second activity session. Participants ranked the methods after they had watched all the demonstration videos or used all the methods. Results from Friedman rank sum tests to compare the before and after-task differences in perceived difficulty, helpfulness and confidence ranks of the four methods are included, so are the *P*-values of the Wilcoxon signed-rank tests that test the ranking differences between the video watching and hands-on sessions.

**Table 6.** Mean ranks of perceived difficulty, helpfulness and confidence level of the four methods and the differences in rankings between the first and the second activity sessions

	Difficulty level rank (1: easiest, 4: hardest)			Helpfulness level rank (1: least helpful, 4: most helpful)			Confidence level rank (1: not at all confident, 5: completely confident)		
Method	Session 1	Session 2	Wilcoxon *P*-value^a^	Session 1	Session 2	Wilcoxon *P*-value	Session 1	Session 2	Wilcoxon *P*-value^a^
Quick Form	1.48	1.24	**0.030**	2.64	2.61	0.769	3.73	4.33	**<0.001**
Wizard	1.88	2.24	**0.029**	2.42	2.09	0.272	3.61	3.67	0.347
Wikidata	3.39	3.60	0.175	2.58	2.73	0.697	2.39	2.30	0.720
Web Protégé	3.24	2.90	**0.031**	2.36	2.58	0.716	2.55	2.97	**0.038**
Friedman *P*-value^b^	**<0.001**	**<0.001**		0.816	0.197		**<0.001**	**<0.001**	

a *P*-value was from Wilcoxon signed-rank test for testing the difference between activity Session 1 and Session 2 in difficulty level rank, helpfulness level rank and confidence level rank.

b *P*-values of the Friedman rank sum test on the differences in perceived difficulty, helpfulness and confidence ranks of the four methods in two sessions.

Values shown in bold indicate statistically significant tests.

Table 6 shows that the four methods were ranked similarly by the participants in both sessions in terms of their helpfulness in recording term semantics and relationships, but differently in terms of perceived difficulty level of the methods (Friedman, *P* < 0.001) and user confidence level of using the methods (Friedman, *P* < 0.001). It also shows that the rankings in Session 1 and Session 2 are often significantly different (see Wilcoxon *P*-values).

On difficulty level, a set of pairwise Wilcoxon signed-rank tests found that after watching the demonstration videos, participants’ difficulty ranking of the four methods was Quick Form = Wizard < WebProtégé= Wikidata, while after the hands-on exercise, the ranking becomes Quick Form < Wizard < WebProtégé< Wikidata (each < indicates a significant difference with *P* < 0.001). In addition, participants found it significantly easier to rank the methods after the hands-on exercise (Wilcoxon signed-rank tests, all *P* < 0.001).

On helpfulness, the ‘no-difference’ finding can be explained by the fact that participants saw each method had its strengths and weaknesses (see also the ‘Results based on response to the open-ended survey questions’ section).

On confidence level, the findings mirror the perceived difficulty levels of the methods. After watching the demonstration videos, the confidence level rank was Quick Form = Wizard > WebProtégé= Wikidata, while after completing the hands-on task, the difference in mean confidence level became statistically significant among all pairs of the four tools (*P* < 0.001), and the confidence level rank was changed to Quick Form > Wizard > WebProtégé> Wikidata (each > indicates a significant difference with *P* < 0.05). After finishing the hands-on task, participants felt more confident using Quick Form (*P* < 0.001) and WebProtégé (*P* = 0.038).

Spearman’s correlation coefficient analyses showed that there is no statistically significant correlation between the post-task confidence in using Quick Form, Wizard or Wikidata and familiarity with a controlled vocabulary, experience with a controlled vocabulary editor or prior confidence in using a controlled vocabulary editor (Table 7). However, post-task confidence in using WebProtégé was found to be positively correlated with participants’ prior familiarity with controlled vocabularies (correlation = 0.414, *P* = 0.009) and the prior confidence in using a controlled vocabulary editor (correlation = 0.416, *P* = 0.009), although both correlations were not strong. This suggests that prior knowledge of controlled vocabularies or confidence in using a controlled vocabulary editor provided increased confidence in the use of WebProtégé.

**Table 7.** Correlation between confidence using each method and past experience with a controlled vocabulary

Confidence on method	Familiarity with controlled vocabulary	Experience with controlled vocabulary editors	Confidence using controlled vocabulary editors
Wizard	Correlation = 0.157 *P* = 0.346	Correlation = −0.124 *P* = 0.460	Correlation = 0.046 *P* = 0.784
Quick Form	Correlation = −0.004 *P* = 0.983	Correlation = −0.087 *P* = 0.605	Correlation = 0.097 *P* = 0.562
WebProtégé	Correlation = 0.414 ***P* = 0.009**	Correlation = 0.058 *P* = 0.728	Correlation = 0.416 ***P* = 0.009**
Wikidata	Correlation = 0.122 *P* = 0.467	Correlation = −0.143 *P* = 0.393	Correlation = 0.310 *P* = 0.059

Values shown in bold indicate statistically significant tests.

This result makes sense because the user interface of WebProtégé is typical of controlled vocabulary editors designed for professionals, while the interfaces for Quick Form, Wizard and Wikidata are all quite different from a typical controlled vocabulary editor. Past experience of the participants with controlled vocabularies does not affect how they experience the other three interfaces.

In addition, a Fisher’s exact test indicates that there is no statistically significant association between post-task confidence level in using Wikidata and past experience with Wiki (Wiki: *P* > 0.05). We examined the rankings of the one participant who self-rated as a Wiki expert user (Figure 9) and noticed that, after the task, the participant ranked Wikidata as more difficult with reduced confidence, both by two scales (difficulty level increased from 1 to 3, confidence level dropped from 4 to 2). These findings suggest that expert users of Wiki are also facing a learning curve when using Wikidata to add terms to an ontology.

##### Results based on response to the open-ended survey questions

Two open-ended survey questions asked participants to comment on the features associated with the four methods that they liked or disliked (Table 8).

**Table 8.** Top ‘likes’ and ‘dislikes’ on the features of the methods based on the two open-ended questions

	Top three features liked by most participants	Top three features disliked by most participants
Quick Form (completely like = 10 completely dislike = 0)	1. Clear instruction. 2. Everything on one page. 3. Minimal data entry.	1. Cannot add other information than what is asked. 2. Should explain ‘taxon’.
Wizard (completely like = 7 completely dislike = 1)	1. Provides guidance and is intuitive. 2. Can answer a question in full without leaving the page. 3. Shows progress information and final summary of axioms added.	1. Limited customization. 2. Cannot go back to previous steps. 3. Find term in a long list can be difficult.
Wikidata (completely like = 0 completely dislike = 4)	1. Terms can have rich connections with other terms. 2. Filter search boxes make it easy to find existing terms/properties. 3. Wiki interface is familiar to users.	1. Complicated and not intuitive. 2. Relating to a new term requires you to leave the page and create the term elsewhere, too many back and forth. 3. Too many properties to choose from. Need more CV Controlled Vocabulary knowledge.
WebProtégé (completely like = 7 completely dislike = 1)	1. Clear, non-scroll UI layout. Shows completed class hierarchy and term information. 2. Filter search boxes make it easy to find existing terms/properties. 3. Relating to a new term is easy: new term auto-added without leaving the page.	1. Not intuitive without instructions. 2. Need more knowledge about CV to use the tool.

### Results from think-aloud session with botanists

The think-aloud session with botanists revealed similar preferences but new interesting observations. Botanists agreed that Wikidata was the most challenging method for ordinary botanists. Our on-site observation confirmed that the botanist who operated the computer completed the task using the three other methods but aborted Wikidata midway.

While agreeing that Quick Form was the easiest method, the botanists recognized that it did not record computer-interpretable semantics of all relationships and properties for a term. Lumping all relevant information into the ‘definition’ box would not provide computers the semantic relations necessary to make the data immediately computable. Consequently, post hoc editing would be necessary to put the data into a computer consumable format.

While participants in the controlled experiment liked Wizard, botanists found the questions asked by Wizard were too detailed and could lead to the addition of erroneous relations and terms to the ontology. They suggested that questions asked by Wizard should be carefully reviewed and that consideration be made to limit the options given to the user.

All three botanists gave WebProtégé a warm welcome when the interface was put on the projector. They spotted the class hierarchy (Figure 5, left side) and commented ‘that list of terms put me right at home’. They did not feel that they had the skills to operate in the WebProtégé environment right away, but believed if they were committed to a role (e.g. community ontology caregiver) that would require them to work with WebProtégé, they were capable of learning it in a short amount of time. They did not think they could say the same for Wikidata.

### Discussion

Quick Form was preferred by student participants and scored with the highest efficiency, lowest difficulty and the highest confidence level before and after the hands-on task. Moreover, the hands-on task reinforced students’ perception of the user-friendliness of the method. From Table 8, we can tell that this high usability stems from a simple, one-page, interface, where it is clear to the users what type of information should be entered into each field. Being able to complete the task or a subtask without leaving the page was also cited as a preferred feature for WebProtégé and Wizard, and the lack of this feature was cited as a ‘dislike’ for Wikidata. Hence, ‘do not break up the user’s workflow’ is a critical feature we need to keep in future design iterations. Other features liked by many include filter search boxes that retrieve matching terms and properties while the user is typing (WebProtégé and Wizard) and the class hierarchy of the terms that shows a whole picture of the ontology (WebProtégé).

While ‘intuitive’ was cited as a ‘like’ by many participants for Quick Form (30 participants) and Wizard (20), participants and botanists also identified a major weakness of the method: the simple form and rigid question sequence does not allow the input of other richer information about a term. In particular, QuickForm provides the least amount of information for constructing semantic-rich ontologies for FAIR data. Participants cited the ability of Wikidata and WebProtégé to accommodate more information about a term and to relate the term to many other terms as a strength of these methods. Some participants recognized the trade-off between a beginner-friendly user interface (e.g. Quick Form and Wizard) where users can effortlessly add basic information about a term to the ontology and a powerful system (e.g. Wikidata and WebProtégé) where untrained users may struggle. For our goal of enabling ordinary biology authors to add terms and relations to their community ontologies, the quantitative and qualitative findings from this study (e.g. Figures 9–11) will help us strike a balance between the two.

Participants commented that more knowledge on controlled vocabularies and ontologies is needed to use WebProtégé or Wikidata more effectively. While both methods support user-defined properties, Wikidata’s complex workflow for adding a term (users have to leave the current page to create a related term on a different page) contributed to many dislikes: 19 out of 34 participants thought Wikidata was complicated and unintuitive; four participants stated that they ‘liked nothing’ about Wikidata and no participant liked it completely. This was the worst score among the four methods (Table 8). In contrast, WebProtégé received comments such as ‘nothing I didn’t like’ from seven participants and only one participant ‘liked nothing’ about WebProtégé. This score is tied with that for Wizard, although Wizard had significantly stronger effectiveness on the specific task than all other methods (Table 4, Figure 11).

It was surprising that both student participants and botanists liked WebProtégé significantly more than Wikidata, and they were more productive when using WebProtégé (Figure 9). We had hypothesized that Wikidata would be preferred because many had used a wiki before. Although participants did cite the familiar wiki interface of Wikidata as a strength, when adding terms to an ontology, they found the process of using Wikidata very complicated. This shows that Wikidata can be a powerful tool, but it also presents a challenge for new users to select the right terms and properties. Even the Wiki expert became less confident with the method after the hands-on task. In contrast, participants became significantly more confident in using WebProtégé after the hands-on task (Table 6).

Our findings conform to some previous studies on the wizard-based user interface, Wikidata and WebProtégé. Babich (2017) suggested the guidance provided by wizards is a major strength, but advanced users may find them inflexible.

In a systematic review of 67 published research articles on Wikidata, Farda-Sarbas and Müller-Birn (25) pointed out that, although many studies on different aspects of Wikidata have been published due to the increasing popularity of the platform, little usability research for Wikidata has been done, especially on the learnability of Wikidata. From Farda-Sarbas and Müller-Birn’s own experiences on conducting Wikidata workshops, ‘it can be said, that people struggle with understanding Wikidata’s central concepts It seems that Wikidata has still untapped potential in becoming accessible for non-technical experts’. Spitz *et al.*  (26) identified a set of key challenges they faced in extracting structured data hosted in Wikidata for their machine learning research. The need for a clear and internally consistent class hierarchy and the need for support for browsing both hierarchies as well as contained items within a hierarchy were identified as two key issues challenging computer algorithms as data consumers. We would further point out that these issues, in addition to several others identified above, also challenge novice human users as data inputters.

When WebProtégé was first introduced, Tudorache *et al.* (27) reported a survey-based usability study involving 13 content experts and classification experts, with or without training in using the tool. The goal was to gauge if the tool was easy to use for domain experts and to identify features that were liked or desired by the users. Responses from domain experts were mixed with equal number of users finding the tool easy to use, difficult to use or natural. Half of the survey participants found the tool too complex and needed support to use it. Our study put WebProtégé side by side with other methods and provided quantitative evidence on the effectiveness, efficiency and user satisfaction with the tool.

### Related work

Building ontologies is complex and time-consuming, and it is even more so if there are various ontology building tools with different functionalities incorporated into the ontology lifecycle (creation, population, validation, deployment, maintenance and evolution). To ease this difficulty, research has been done to determine the advantages and disadvantages of the tools and their suitability for various ontologies and user groups.

One approach to tool evaluation is feature-based comparative studies, where features or functionalities of tools are examined by experts (e.g. 28, 29, 30) or through surveys (e.g. (32), Khondoker and Mueller, (2010 a b); Youn and Arora 2009). Such studies do not evaluate users’ experience with tools but evaluate whether a tool has certain features or not. For instance, Denny (34) compared over 50 ontology development tools by checking whether they have certain features and describing them (e.g. modeling features and limitations, base language, web support and use, import/export format, graph view, consistency checks, multiuser support, merging, lexical support and information extraction). Youn and Arora (2009) compared 14 editors along with their import format, export format, graph view, consistency check, multiuser support, Web support, merging function, collaborative work support, ontology library, inference engine, exception handling, ontology storage, extensibility and availability. Kapoor and Sharma (28) reviewed open source editors Protégé 3.4, Apollo, IsaViz & SWOOP in terms of interoperability, openness, easiness to update and maintain, market status and penetration. Ranpara *et al.* (29) compared the same four tools in the context of cross-platform integration, easy to update and manage, and tolerance. In addition to the above features, a semiotic framework for understanding the quality of conceptual modeling was used to establish the features to be compared in Su and Ilebrekke (30). The features identified overlap with features used in the other studies described above. Feature comparisons provide useful guidance for tool selection; however, without involving end users in the evaluation, it is unclear if the features are effective, efficient and pleasant to use. In other words, would people actually use the tool, and would the tool achieve its purpose.

**Figure 14.**
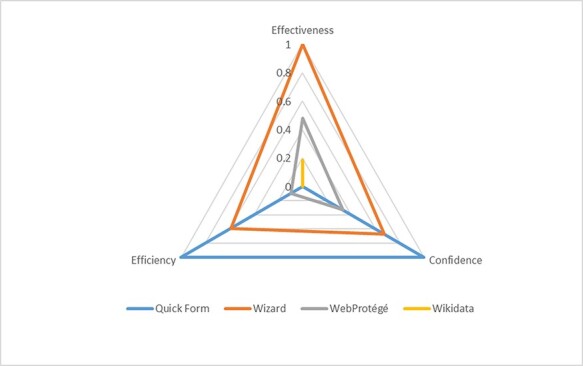
Efficiency (cost)-effectiveness and user satisfaction ranking of the four methods.

There are a few studies that evaluate ontology building tools based on user experience. Khondoker and Mueller, (2010 a b) (32) conducted an online survey to understand which tools are most frequently used by users and their drawbacks. Thirty-two ontology engineers responded to the survey, with the most popular tool being Protégé (now TopBraid is quickly catching up). User attitude and satisfaction ratings with Protégé were analyzed, along with comments from users with a range of experience with the tool. The results suggested that ontology engineers found Protégé was a good tool for ontology development, despite an initial learning curve.

Several hands-on experiments related to ontology visualization have been reported. A controlled usability study was reported by Fu *et al.* (35) to compare two visuals (indented tree vs. graph) for ontology mapping with 36 novice users via a hands-on task. Their evaluation metrics include effectiveness, efficiency, workload and satisfaction of these two techniques. Katifori *et al.* (36) evaluated four different visualization methods in Protégé with 37 participants from a university using a few information retrieval tasks in which ontologies were used as browsing aids. These four ontology visualization methods are evaluated based on the correct answer percentages, comparative measured times and user comments.

Closest to our study are that by García-Barriocanal *et al.* (24), who carried out a usability evaluation of four ontology editors (Protégé 2000, OntoEdit 2.0, OILEd, KSL Ontology Editor) by first conducting an expert heuristic pre-assessment of the tools, followed by user experiments to further investigate identified issues. The later phase of the research involved three groups of participants with different levels of concept modeling skills completing an ontology creating task and an ontology updating task. Each group had four participants. Owing to the small sample size in each group, only averages of group means were reported on task completion time and user error counts were analyzed without statistical tests. They concluded that the GUI-desktop-base ontology editors tested did not possess major usability issues and were fairly adequate for new users. All four editors evaluated in this study are more or less similar to Protégé or WebProtégé as they were all designed for professional ontology developers. In our study, we compared WebProtégé with three other very different methods with a larger number of participants, which allowed us to identify significant differences among the methods.

### Conclusion and future work

We conducted two usability evaluations on four selected methods for adding terms to an ontology with the goal of identifying good design elements for the author-driven FAIR data production system (Figure 1). The four methods that were compared represent different approaches an ordinary biology author may use to add new terms to their community ontologies. Materials used in the usability evaluations are included in the Appendix.

Our findings provide a rather clear picture of the effectiveness, efficiency and user satisfaction among the four methods. Integrating the results presented in Table 6, Figures 9 and 12, we can plot the relationships between these parameters for the four methods as a radar plot (Figure 14), after a 0–1 normalization of their respective scores. Each triangle in the plot represents one method (Wikidata’s triangle collapsed into a line segment). Quick Form has low effectiveness (i.e. is the least FAIR), high efficiency (low cost) and high confidence. Wizard has high effectiveness, medium efficiency (medium cost) and medium confidence. WebProtégé has medium effectiveness, medium efficiency (medium cost) and medium confidence. Wikidata has relatively low effectiveness, low confidence and low efficiency (high cost). The areas included in triangles intuitively represent the overall usability of the four methods for this group of users, when effectiveness, efficiency and confidence are weighted equally: Wizard is the best, closely followed by Quick Form, then by WebProtégé and last Wikidata. Wikidata is a rich knowledge base and has its unique strength in its powerful APIs, but our finding suggests that it is not user-friendly when used by a human user to add or edit terms.

Based on our findings, we will implement modified versions of Quick Form and Wizard in our author-driven FAIR data production system for their high user confidence and balanced cost-effectiveness and user confidence. We will employ filter search boxes and include complete term and property hierarchies as appropriate in those user interfaces. We will design a simplified version of Wizard for various ontology design patterns, which are effective patterns for modeling specific knowledge points, involving the addition of a set of axioms Wizard will ensure all axioms are added for a specific pattern. We will also train a few super users in ontology knowledge so they can employ WebProtégé to resolve potential conflicts in user input to the ontology. Wikidata is a power knowledge base that supports cross-references among terms, but it is not an effective nor easy tool for authors to use.

### Funding

This material is based upon work supported by the National Science Foundation under Grant No. 1661485. Any opinions, findings, and conclusions or recommendations expressed in this material are those of the author(s) and do not necessarily reflect the views of the National Science. Foundation

References1.


Cui
H.
, DahdulW., DececchiA.T.  et al. (2015) CharaParser+EQ: performance evaluation without gold standard. *Proc Assoc Inf Sci Technol*, 52, 1–10.2.


Dahdul
W.
, MandaP., CuiH.  et al. (2018) Annotation of phenotypes using ontologies: a gold standard for the training and evaluation of natural language processing systems. *Database*.10.1093/database/bay110PMC6301375305764853.


Wilkinson
M.D.
, DumontierM., AalbersbergI.J.  et al. (2016) The FAIR guiding principles for scientific data management and stewardship. *Sci. Data*, 3, 1–9.10.1038/sdata.2016.18PMC4792175269782444.


Cui
H.
, MacklinJ., SachsJ.  et al. (2018) Incentivising use of structured language in biological descriptions: author-driven phenotype data and ontology production. *Biodivers Data J*, 6, e29616.10.3897/BDJ.6.e29616PMC6235995304736205.


Smith
B.
, AshburnerM., RosseC.  et al. (2007) The OBO foundry: coordinated evolution of ontologies to support biomedical data integration. *Nat. Biotechnol.*, 25, 1251.10.1038/nbt1346PMC2814061179896876.


Ongenae
F.
, DuysburghP., SulmonN.  et al. (2014) An ontology co-design method for the co-creation of a continuous care ontology. *Appl Ontol*, 9, 27–64.7.


Brooke
J.

 (1996) *SUS - A Quick and Dirty Usability Scale*. Redhatch Consulting Ltd, Earley, Reading, p. 7.8.


Dumas
J.S.
, DumasJ.S. and RedishJ. (1999) *A Practical Guide to Usability Testing*. Intellect books. Fishponds, Bristol.9.


Nielsen
J.

 (1993) Iterative user-interface design. *Computer*, 26, 32–41.10.


Preece
J.

 (2000) *Online Communities: Designing Usability and Supporting Sociability*. John Wiley & Sons, Inc, Chichester, England.11.


Nielsen
J.

 (1994) Usability laboratories. *Behav Inf Technol*, 13, 3–8.12.


Musen
M.A.

 (2015) The protégé project: a look back and a look forward. *AI Matters*, 1, 4–12.2723955610.1145/2757001.2757003PMC488368413.


Tudorache
T.
, VendettiJ. and NoyN. (2008) Web-protege: a lightweight OWL ontology editor for the web. In: Fifth OWLED Workshop on OWL: Experiences and Directions Seventh International Semantic Web Conference (ISWC-2008), Karlsruhe, Germany.14.


Babich
N.

 (2017). *Wizard Design Pattern*. Medium. https://uxplanet.org/wizard-design-pattern-8c86e14f2a38 (21 July 2020, date last accessed).15.Wikidata (2021) https://www.wikidata.org/wiki/Wikidata:Main_Page (31 May 2021, date last accessed).16.


Braun
S.
, SchmidtA., WalterA.  et al. (2007) *Ontology Maturing: A Collaborative Web 2.0 Approach to Ontology Engineering*World Wide Web Conference (WWW 07), Banff, Canada.17.


Piscopo
A.
 and SimperlE. (2018) Who models the world? Collaborative ontology creation and user roles in Wikidata. *Proc ACM Hum Comput Interact*, 2, 118.18.


Vrandečić
D.
 and KrötzschM. (2014) Wikidata: a free collaborative knowledgebase. *Commun ACM*, 57, 78–85.19.


Horridge
M.
, TudoracheT., NuylasC.  et al. (2014) WebProtégé: A collaborative web-based platform for editing biomedical ontologies. *Bioinformatics*, 30, 2384–2385.2477156010.1093/bioinformatics/btu256PMC417605720.


Noy
N.F.
, SintekM., DeckerS.  et al. (2001) Creating semantic web contents with protege-2000. *IEEE Intell Syst*, 16, 60–71.21.


Cui  H., Zhang  L., Ford  B.  et al. (2020) Measurement recorder: developing a useful tool for making species descriptions that produces computable phenotypes. Database, 2020doi: 10.1093/database/baaa079.PMC76787893321689622.

ISO 9241-11:2018(en)
. (2018) *Ergonomics of human-system interaction—Part 11: Usability: Definitions and concepts*. https://www.iso.org/obp/ui/#iso:std:iso:9241:-11:ed-2:v1:en (21 July 2020, date last accessed).23.


Khondoker
M.R.
 and MuellerP. (2010). Comparing ontology development tools based on an online survey. In: Proceedings of the World Congress on Engineering 2010 Vol I WCE 2010, June 30 - July 2, 2010, London, U.K, p. 6.24.


García-Barriocanal
E.
, SiciliaM.A. and Sánchez-AlonsoS. (2005) Usability Evaluation of Ontology Editors. *KO knowl organ*, 32, 1–9.25.


Farda-Sarbas
M.
 and Müller-BirnC. (2019) *Wikidata from a*  *Research Perspective**—**A Systematic Mapping Study*  *of*  *Wikidata*. ArXiv:1908.11153 [Cs]. http://arxiv.org/abs/1908.11153 (21 July 2020, date last accessed).26.


Spitz
A.
, DixitV., RichterL.  et al. (2016). State of the union: A data consumer’s perspective on Wikidata and its properties for the classification and resolution of entities. In: Tenth International AAAI Conference on Web and Social Media. Tenth International AAAI Conference on Web and Social Media, Cologne, Germany. https://www.aaai.org/ocs/index.php/ICWSM/ICWSM16/paper/view/13200 (21 July 2020, date last accessed).27.


Tudorache
T.


Falconer
S.


Nyulas
C.

  et al. (2010) Will semantic web technologies work for the development of ICD-11? In: Patel-Schneider  PF, Pan  Y, Hitzler  P  et al. (ed.) *The Semantic Web – ISWC 2010. ISWC 2010. Lecture Notes in Computer Science*. Springer, Berlin, Heidelberg. Vol. 6497 pp. 257–272.28.


Kapoor
B.
 and SharmaS. (2010) A comparative study ontology building tools for semantic web applications. *Int J Web and Semantic Technol*, 1, 1–13.29.


Ranpara
R.


Yusufzai
A.


Kumbharana
C.K.

 (2019) A comparative study of ontology building tools for contextual information retrieval. In: Rathore  VS, Worring  M, Mishra  DK  *et al*. (eds). *Emerging Trends in Expert Applications and Security*. Springer, Singapore, pp. 401–408.30.


Su
X.


Ilebrekke
L.

 (2002) A comparative study of ontology languages and tools. In: Pidduck  AB, Ozsu  MT, Mylopoulos  J  *et al*. (eds). *Advanced Information Systems Engineering*. Springer, Berlin, Heidelberg, pp. 761–765.31.


Norta
A.
, YangarberR. and CarlsonL. (2010). Utility evaluation of tools for collaborative development and maintenance of ontologies. In: 2010 14th IEEE International Enterprise Distributed Object Computing Conference Workshops, IEEE, Vitoria, Brazil, pp. 207–214.32.


Khondoker  P. M.
, (2010)“Comparing Ontology Development Tools Based on an Online Survey”. World Congress on Engineering, LondonWorld Congress on Engineering, London, UK, pp. 1299–1313.33.


Youn  S., Arora  A., Ch  P.  et al. (2009) *Survey about Ontology Development Tools for Ontology-based Knowledge Management*. University of South California, CA, USA. http://wwwscf.usc.edu/csci586/projects/. (25 November 2005, date last accessed).34.


Denny
M.

 (2002). Ontology Building: a Survey of Editing Tools. https://www.xml.com/pub/a/2002/11/06/ontologies.html (21 July 2020, date last accessed).35.


Fu
B.


Noy
N.F.


Storey
M.-A.

 (2013) Indented tree or graph? A usability study of ontology visualization techniques in the context of class mapping evaluation. In: Alani  H, Kagal  L, Fokoue  A  *et al.* (eds). *The Semantic Web – ISWC 2013*. Springer, Berlin, Heidelberg, pp. 117–134.36.


Katifori
A.
, TorouE., VassilakisC.  et al. (2008). Selected results of a comparative study of four ontology visualization methods for information retrieval tasks. In: 2008 Second International Conference on Research Challenges in Information Science, IEEE, Marrakech, Morocco, pp. 133–140.37.


Casellas
N.

 (2009) Ontology evaluation through usability measures. In: Meersman  R, Herrero  P, Dillon  T (eds). *On the Move to Meaningful Internet Systems: OTM 2009 Workshops*. Springer, Berlin, Heidelberg, pp. 594–603.38.


Grafkin
P.
, MironovM., FellmannM.  et al. (2016) *SPARQL Query Builders: Overview and Comparison* Prague, Czech Republic,. p. 12.39.

The Plant Ontology Consortium
 (2002) The plant ontology ^TM^ consortium and plant otologies. Comp. Funct. Genomics, 3, 137–142.1862884210.1002/cfg.154PMC244726340.


Yoder
M.J.
, MikóI., SeltmannK.C.  et al. (2010) A gross anatomy ontology for hymenoptera. *PLoS One*, 5, e15991.10.1371/journal.pone.0015991PMC301212321209921
